# Malarial Fevers—Cause and Treatment

**Published:** 1877-11-20

**Authors:** J. M. Lewis

**Affiliations:** Kosciusko, Miss.


### SOUTHERN MEDICAL RECORD.

VOL. VII. ATLANTA, GA., NOVEMBER 20, 1877. No. 11.

TOMAS S. POWELL, M.D.,

W. T. GOLDSMITH, M. D., } EDITORS.

R. C. WORD, M. D.,

R. C. WORD, Business Manager.

SUBSCRIPTION: TWO DOLLARS PER ANNUM, IN ADVANCE.

☞ All communications, and letters on business connected with THE RECORD for 1877 must be addressed to the Business Manager.

### Original and Selected Articles.

### MALARIAL FEVERS—CAUSE AND TREATMENT.

By J. M. LEWIS, M.D., Kosciusko, Miss.

It may be asked if I have anything new to offer on this subject. If the question refers to what is known in reference to "malaria," I would answer, no; but if to the treatment of the fevers, as generally adopted by the physicians of this country, I would answer, yes.

I fear that, too often, in our communications to the journals, we prefer to report novel cases, instead of our experience with familiar diseases. Certainly we in this country cannot write or speak too often about malaria, when we consider at least one-third of our practice is dealing with this poison. In order to speak of the treatment, which is the main object of this paper, I will be allowed to quote from some of our best authorities in reference to what "malaria" is.

Niemyer says: "I have no hesitation in saying, decidedly, that marsh miasm—malaria—must consist of low vegetable organisms, whose development is chiefly due to the putrefaction of vegetable substances. It is true, these low organisms have not actually been observed: no one has seen '*malarious spores.*'

"This poison arises from marshy land in particular conditions, such as decomposition under the influence of partial moisture, and of heat above 60° Fah. If the land is perfectly dry, or perfectly flooded, or frozen, the poison is not generated. It is believed to be a material poison. It may be wafted along with the wind, and so induce fever at a distance from the place where the poison is generated. It may also be intercepted by a belt of trees."

Aitken says: "In these forms of fevers, (speaking of intermittent, remittent and malarious forms of yellow fever), a malarious poison of an unknown kind, generated in paludal regions or littoral districts, is absorbed and affects the blood, as *cholera*, *typhus*, and other '*miasmatic*' poisons do. The poison, in the absence of any better name, is known as '*malaria.*'"

Flint, Wood, and others, say virtually the same.

From the above, it will be conceded that "malaria" is a poison, generated under certain circumstances, a "miasm"

produced, perhaps, by the decomposition of vegetable matter, heat and moisture being required.

This poison being absorbed causes a variety of fevers, which we call intermittent, remittent, malarial, hemorrhagic, and malarial yellow fevers, all these occurring in different degrees of intensity, from a mild intermittent to a pernicious remittent.

Alison says: "If any one has seen only the milder forms of remittent fevers, and had no opportunity of tracing up their several grades, he might well believe, when he saw suddenly the severest variety, that he had before him a distinct affection."

Aitken says (refering to above fevers), all are "similar pathological," *i.e.*, all caused from a malarial poison.

Why the same poison will cause an intermittent fever in one person and remittent fever in another, both being exposed alike, (which we often observe,) is something that we cannot explain; but that it is one and the same poison we have every reason to believe. I will not speak of the diagnosis of these fevers, for they will be easily recognized by their periodicity, and are fully described in our text books on practice.

TREATMENT.—For some time I have had but one remedy for these fevers—and that is sulphate of quinine. I am satisfied all other remedies, (or at least the most of them) are hurtful, especially the one most used, viz: calomel. Mercury in any form is certainly adding fuel to the fire, and not only does harm at the time, but prolongs convalesence.

Headland says: "Mercury excites the functions of the liver, and bowels, being both cathartic and chologogue." Is not the liver in these fevers already excited to increased action, pouring out bile into the stomach and bowels? Why then impose on the abused organ? If we had a remedy that would suspend for a time, the secretions of the liver, would it not be more rational to give it? Mercury was given years ago, and is administered yet by some, (at least two-thirds who practice in this country) under the impression that these fevers were inflammatory. With this idea of the Pathology, there might be some excuse for the practice, but as shown in the first part of the paper, malaria is a poison, affecting every organ in the body, and can be quickly (in the majority of cases) arrested by the use of quinine.

Mercury is one of our most debilitating remedies. "It decomposes the *blood*, and by some destructive agency, it deprives it of one-third of its fibrine, one-seventh of its albumen, one-sixth, or more of its globules, and at the same time loads it with a fœtid matter, the products of decomposition. Such power is posessed by few other medicines, and certainly exerted by none in the same degree as mercury. It is an agent of terrible activity, and we may well be cautious how we handle it. *It disintegrates or decomposes the blood, and thus wastes the body.*

After using mercury physicians speak of the dark offensive discharge that takes place, calling it *bile*, when it owes its color and offensiveness to the decomposition of the mercury in the system, forming a sub-sulphide. The same result can be had in a healthy man, by the use of mercury. The mercury that is given (in many cases) would result more disastrously than it does, were it not for the fact that our systems only absorb so much of mercury, the balance passing through as inert matter.

I believe many cases of reported failures to effect a cure with quinine, are the result of prolonged treatment with mercury. This idea of ("preparatory treatment") first giving calomel and wait until it acts on the bowels, and the fever cools, before using quinine, is deeply rooted in the minds of the people. A farmer said to me a short time ago: "He knew quinine was *the remedy*, but thought that calomel had to be administered first, in order for the quinine to have its good effects." There are many men practicing medicine in this country to-day, who hold to the same idea. Many cases, no doubt, are annually lost by not giving quinine freely as soon as the patient is seen. This is the practice of Prof. Flint, of New York, and he says he has advocated it for thirty years. In his excellent work on practice, he says:

"For the cure of intermittent fever, medicine possesses specifics, if any remedies are entitled to this appellation. This statement applies especially to the salts of quinia, of which the sulphate is the one almost universally used. The sulphate of quinia will promptly interrupt the recurrence of the paroxysms of intermittent fever in the vast majority of cases. It is always desirable to arrest the disease as speedily as possible. There is no need of preparatory treatment. The use of mercurial cathartics, emetics and bleeding, are injurious, and are not indicated. The pathological views which formerly led practitioners to employ mercury freely in this disease, (speaking of remittent fevers) are not *tenable*, and it may be fairly doubted if clinical observation afford any ground for regarding this remedy as specially indicated."

I have not had the experience that Prof. Flint has, and do not speak with as much authority, as he was practicing before I was born; but so far as it goes I am fully satisfied with quinine alone in the treatment of malarial fevers. To those who adopt similar practice, and they are many, this paper is worthless, but I will venture you have a brother practitioner, who enjoys the confidence of the community in which he lives, to as great an extent as you do—who adopts the old practice—calomel, calomel and quinine, and then quinine and calomel, and when his patient comes back, after he gets through with the above, mouth sore, body wasted, pale and thin, more from the effects of the mercury given than the disease, he will say, "John, you've had a hard spell."

I have seen many cases of fever in the last few days (and at this season of the year, September 1st, we have many cases along the swamp, a few miles from town), when the patient had high fever, delirious, complaining of intense pain in the head, bones aching, restless, etc., and, in little children, convulsions, result of fever, when I would administer, to an adult, 15 to 20 grains of quinine (children's dose in proportion to age), and, if their stomach rejects it, which it will do in many instances, give the same quantity by enema, and often in an hour's time the patient will become rational, fever begin to cool (skin moist), pulse reduced, etc.; and, if it be a child, with convulsions, they will cease. The effect of the remedy is truly wonderful. I will not speak of my own experience, for fear my readers may think I am disposed to boast, but when I am called to a malarial fever case, be it ever so slight, I always give quinine actively, for you cannot tell but what it will assume the pernicious form in a few hours. By this mode of treatment the mild cases will be promptly arrested, and the severe ones will be checked.

Of late I have used sulphate of cinchonidia as a substitute for quinine, and am well pleased with the results.

